# Commentary: Molecular characterization of *Smtdc-1* and *Smddc-1* discloses roles as male-competence factors for the sexual maturation of *Schistosoma mansoni* females

**DOI:** 10.3389/fcimb.2025.1564081

**Published:** 2025-04-09

**Authors:** Haoran Zhong, Yamei Jin

**Affiliations:** National Reference Laboratory for Animal Schistosomiasis, Key Laboratory of Animal Parasitology of Ministry of Agriculture and Rural Affairs, Shanghai Veterinary Research Institute, Chinese Academy of Agricultural Sciences, Shanghai, China

**Keywords:** schistosome, reproduction, helminth, development, dopa decarboxylase (DDC)

## Introduction

Schistosomiasis, also known as bilharzia, is a neglected tropical disease caused by parasitic flatworms of the genus *Schistosoma*, predominantly affecting impoverished communities in tropical and subtropical regions (Lo et al., 2022). Within the host, adult schistosomes reside in blood vessels, where their unique dioecious nature plays a critical role in disease progression. Male schistosomes use their specialized gynecophoral canal to cradle females, maintaining constant physical pairing that is essential for the development of females to adulthood and the maturation of their reproductive organs, leading to egg production (Nation et al., 2020). These eggs, the primary drivers of pathology, can become lodged in host tissues, triggering granulomatous inflammation and severe complications such as periportal fibrosis, portal hypertension, and urogenital lesions (McManus et al., 2018). Elucidating the molecular mechanisms underlying male–female interactions in schistosomes holds significant potential for developing innovative strategies to disrupt reproduction and curb disease transmission.

## General comments

In a recent study entitled “Molecular characterization of *Smtdc-1* and *Smddc-1* discloses roles as male-competence factors for the sexual maturation of *Schistosoma mansoni* females” was published in Frontiers in Cellular and Infection Microbiology. Li et al. investigated the roles of tyrosine decarboxylase (TDC) and DOPA decarboxylase (DDC) as critical male-specific or male-preferential factors in facilitating the sexual maturation of female *S. mansoni*. Using RNA interference (RNAi), the authors provided compelling evidence that these male-biased, pairing-dependent genes influence oocyte differentiation and egg production in females, thereby offering valuable insights into the reproductive biology of *Schistosoma*. Additionally, the study utilized an improved culture medium that substituted human low-density lipoprotein (LDL) for porcine cholesterol, enhancing the stability and re-pairing efficiency of male and female schistosomes *in vitro*. This new medium formulation may serve as a valuable tool for future studies on male–female pairing processes. We greatly appreciate this work and recognize its significant contribution. However, we need to point out that the study relies heavily on indirect evidence and lacks additional experimental approaches to definitively establish the direct roles of DDC and TDC in male–female communication.

First, focusing solely on the morphological changes in the testes following RNAi is insufficient to assess the biological function of these genes/proteins in male schistosomes. Based on the whole-mount *in situ* hybridization localization image presented in this study, DDC and TDC show weak signals in the testes, indicating that they are not highly expressed in this tissue and may not directly contribute to testicular biological functions. This observation may explain the lack of significant morphological alterations observed following gene knockdown. Similar localization patterns have also been observed in *Schistosoma japonicum* (Wang et al., 2017), further suggesting that DDC and TDC may not play direct testicular biological function in testes. Consequently, the appearance of the testes should not be considered the sole indicator of male functionality. Given that these genes are highly expressed in neurons, it would be more informative to assess neuronal morphology in RNAi-treated males. Disrupted neuronal function could impact male and female interaction, which are essential for female reproductive development. Current research suggests that male schistosomes stimulate female reproductive development by secreting signaling molecules that influence female physiology. Therefore, it is essential to assess the secretion of biogenic amines or the overall secretory capacity of the male schistosomes to determine whether the knockdown of DDC and TDC impacts male signaling functions that are critical for female development (Chen et al., 2022).

Second, while the RNAi experiments demonstrate functional relevance, the specific metabolic products catalyzed by DDC and TDC in schistosome remain unidentified, leaving it unclear whether these metabolites directly regulate female reproductive processes or act through secondary pathways. Among these potential products, β-alanyl-tryptamine (BATT), a nonribosomal dipeptide, has been identified as a crucial signaling molecule in *S.mansoni*, playing an essential role in male-mediated regulation of female reproductive development (Chen et al., 2022). In planarian studies, researchers found that DDC is critical for the synthesis of BATT, catalyzing the conversion of tryptophan into tryptamine, which is subsequently conjugated with β-alanine by nonribosomal peptide synthetase (NRPS) to form BATT (Issigonis et al., 2024). Supplementation with BATT successfully reversed developmental defects caused by DDC knockdown, underscoring the importance of DDC’s enzymatic activity as a key upstream step in BATT production. This pathway highlights the potential of metabolic rescue approaches not only to validate DDC’s role but also to elucidate its connection to reproductive biology. A promising direction for future studies would be to perform metabolic profiling of RNAi-treated males, which could reveal whether additional metabolites are altered in the absence of these gene products. Applying such a metabolic profiling approach in *Schistosoma* could provide direct evidence for DDC’s function and further clarify its contributions to the parasite’s reproductive system.

However, this approach may face unique challenges in schistosomes due to their more complex life cycle, physiology, and host-dependent reproductive system (Wang et al., 2019). Although BATT supplementation has been shown to stimulate female reproductive development in schistosomes, the developmental state achieved remains inferior to that of naturally paired parasites in the host (Chen et al., 2022), suggesting that additional factors—potentially including DDC-derived metabolites—are involved in optimal reproductive development. Furthermore, host-derived nutrients are also crucial in the reproductive biology of schistosomes, and exploring the interaction between host-provided resources, is an important avenue for future research.

## Discussion

We highly appreciate the work of Li et al. on DDC and TDC, which provides valuable insights into the roles of these enzymes in reproductive biology and serves as a significant reference for understanding the evolution and reproductive development of flatworms. Their findings shed light on the molecular mechanisms underlying male–female communication in *S. mansoni*. However, to definitively establish the direct roles of DDC and TDC, further experimental validation is essential. Metabolic rescue approaches and identification of specific metabolites could strengthen the evidence and clarify their precise contributions to the reproductive processes of this parasitic species.
